# Molecular phylogenetic and morphometric analysis of population structure and demography of endangered threadfin fish *Eleutheronema* from Indo-Pacific waters

**DOI:** 10.1038/s41598-022-07342-w

**Published:** 2022-03-02

**Authors:** Jie Xiao, Shaoliang Lyu, Teuku H. Iqbal, Sukree Hajisamae, Karl W. K. Tsim, Wen-Xiong Wang

**Affiliations:** 1 School of Energy and Environment and State Key Laboratory of Marine Pollution, City University of Hong Kong, Kowloon, Hong Kong China; 2 Faculty of Science and Technology, Prince of Songkla University, Pattani, 94000 Thailand; 3 Division of Life Science, Hong Kong University of Science and Technology, Clear Water Bay, Kowloon, Hong Kong China; 4 Research Centre for the Oceans and Human Health, City University of Hong Kong Shenzhen Research Institute, Shenzhen, 518057 China

**Keywords:** Ecology, Environmental sciences, Ocean sciences

## Abstract

The threadfin *Eleutheronema* are the important fishery resources in Indo-Pacific regions and classified as the endangered species with considerable conservation values. Their genetic diversity and population structure remain essentially unknown but are critical for the proper management and sustainable harvests of such important fisheries. Here, the mitochondrial DNA sequences of *CO1* and *16s rRNA* were determined from 75 individuals of *Eleutheronema tetradactylum* and 89 individuals of *Eleutheronema rhadinum* collected from different locations of South China Sea and Thailand coastal waters. Genetic diversity analysis revealed that both *E. tetradactylum* (Haplotype diversity, H = 0.105–0.211; Nucleotide diversity, π = 0.00017–0.00043) and *E. rhadinum* (H = 0.074–0.663, π = 0.00013–0.01088) had low diversity. Population structure analysis demonstrated the shallow genetic differentiation among the South China Sea populations. The limited communication between China and Thailand populations caused the high genetic differentiation in all groups due to the low dispersal ability. Reconstruction of *CO1* phylogenetic relationships and demographic studies across Indo-West-Pacific regions provided strong evidence for a shared common origin or ancestor of *E. tetradactylum* and *E. rhadinum*. *Eleutheronema rhadinum* were further subdivided into two distinct genetic lineages, with Clade A dominantly distributing in Thailand and Malaysia and Clade B distributing in China coastal waters. Phenotypic divergence, characterized mainly by the depth of caudal peduncle and length of caudal peduncle, was also observed for all populations, which was possibly associated with specific local adaptations to environmental changes. Our study suggested a strong need for the development of proper fishery management strategies and conservation actions for the imperiled *Eleutheronema* species.

## Introduction

The genera *Eleutheronema* belongs to the Family Polynemidae, which consists of three valid species, including three fingers threadfin (*Eleutheronema tridactylum*), four fingers threadfin (*Eleutheronema tetradactylum*), and East Asian four fingers threadfin (*Eleutheronema rhadinum*). *Eleutheronema tetradactylum*, also known as the Indian salmon due to its wide distribution in the Indo-West-Pacific region, is an important commercial fish species. However, fisheries of *E. tetradactylum* have drastically decreased over the past decade due to the over-exploitation and possibly water pollution^1,2^. The fish has been classified as endangered by the International Union for Conservation of Nature (IUCN). The East Asian four-finger threadfin, *E. rhadinum*, is endemic to the East Asia region, including China coastal water (South and East China Seas), Japan, Vietnam, and Malaysia^3^. Especially in the China coastal water, it is a commercially crucial fish for coastal and inshore small-scale fisheries, with high auction prices in local fish markets. In contrast, *E. tridactylum* is relatively rare but also considered as a valid species.

*Eleutheronema tridactylum* can be easily distinguished from the other two *Eleutheronema* species by determining the morphological characters such as pectoral fins^1^. However, identification of *E. tetradactylum* and *E. rhadinum* is relatively difficult based on their morphological discrimination by visual check due to their external morphological similarities. Therefore, molecular markers such as mitochondrial DNA sequencing are helpful for accurate species and bloodstock identification^4^. The cytochrome c oxidase subunit 1 (*CO1*) gene has been used to discriminate *Eleutheronema* species, demonstrating the advantages of the molecular marker in identifying and resolving issues in taxonomy and geographical distribution of taxa^3^. In addition, mtDNA, particularly the *CO1* and *16s rRNA* genes, have been proven to be the powerful tools for revealing the phylogeographic patterns and genetic diversity.

The distribution of species is a complex expression of its ecological and evolutionary history, and assessing the population genetic, morphological, and environmental data could provide new insight into the effects of the environment on the population structure of species. A previous study on the population differentiation of *E. rhadinum* in the East and South China Seas regions in 2013 was based on the *CO1* method^5^. The results revealed that the populations from Qingdao, Zhuhai, and Zhoushan developed divergent genetic structures and experienced a population expansion. Wang et al.^6^ used the mtDNA *Cytb* method to analyze the population diversity of *E. tetradactylum* in both East and South China Seas, including Zhoushan, Wenzhou, Shantou, and Qionghai, which showed a high level of haplotype diversity and low nucleotide diversity among three populations.

To date, knowledge of the population differentiation and genetic diversity of *E. tetradactylum* and *E. rhadinum* in Southeastern regions, especially the geographical differentiation, is still unknown. This study analyzed the morphological differentiation and population genetic structure of *Eleutheronema* species with morphometric measurements and *CO1* and *16s rRNA* sequences newly obtained from 75 *E. tetradactylum* and 89 *E. rhadinum* individuals from Southern China and Thailand (west and east) coastal waters. Our study focused on the genetic differences between the China and Thailand populations. The morphological analysis, one of the simplest and most efficient methods to identify fish stock structure and discriminate the species, were conducted by using differences in body measurements and morphological characteristics. We performed the quantitative morphological study to investigate whether allopatric *Eleutheronema* differed morphologically. Additionally, the newly obtained data were subjected for phylogenetic relationships and historical demography analysis of *Eleutheronema* species across the Indo-West-Pacific regions by integrating with previously reported data. Assessing the population diversity and differentiation of *Eleutheronema* species is crucial for evaluating the adaptive ability to the changeable environment and essential for establishing the conservation strategies.

## Materials and methods

### Sample collection and DNA extraction

**Figure 1:**
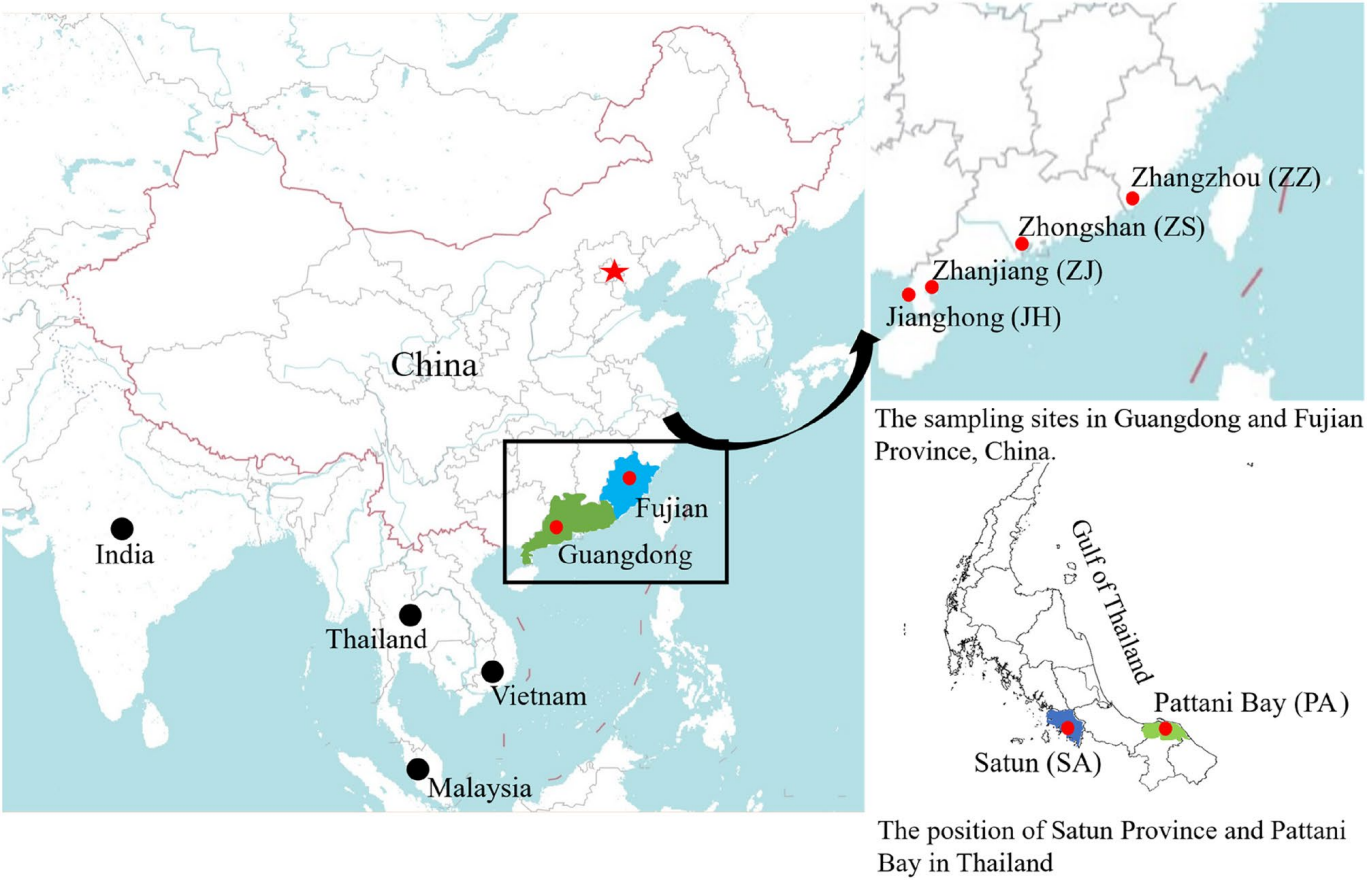
Distribution of sampling locations in the China and Thailand coastal waters. The maps were generated by Adobe Illustrator CC 2020 using a GIS shape file retrived from administrative areas database in DIVA GIS (https://www.diva-gis.org/) and DataV.GeoAtlas (http://guihuayun.com/maps/region_datav.php).

A total of 75 *Eleutheronema tetradactylum* and 89 *Eleutheronema rhadinum* individuals were collected, respectively, from three (Zhongshan and Zhanjiang in China, and Pattani Bay in Thailand) and four (Zhanjiang, Jianghong, and Zhangzhou in China, and Satun in Thailand) sites during Nov. 2020 to Jan. 2021 (Fig. 1, Table S1). Among them, the fishes in Zhongshan were collected from the commercial breeding line (China), whereas all other fishes were wild caught by boats. For all these collected fishes, each individual was firstly photographed with a digital camera, and the photographs were processed for morphological analysis. For molecular analysis, about 100 mg muscle of each individual was clipped, placed in 95% ethanol, and kept at − 20 °C in the laboratory until DNA extraction. Genomic DNA was extracted from each individual using a commercial DNA extraction kit (TIANGEN Biotech) and following the manufacturer’s protocol. All DNA samples were kept at − 80 °C for further phylogenetic analysis.

### PCR amplification and sequencing

To amplify the partial mtDNA fragments of *CO1* (614 bp) and *16s rRNA* (574 bp), PCR was carried out using the previously universal primers for *CO1* and *16s rRNA*^7^. The 25-μL PCR reaction system included 2.5 μL of 10 × Buffer, 2.0 μL of dNTPs, 1.0 μL of the primers (0.5 μL forward and 0.5 μL backward), 0.5 μL of template DNA, 0.2 μL of Taq (TaKaRa), and 18.8 μL of ddH_2_O. The PCR cycle parameters were as follows: 94 °C for 4 min, 30 cycles of 94 °C for 30 s, 50 °C for 30 s, and 72 °C for 45 s, and 72 °C for 5 min for the final extension. One μL of each PCR product was detected by 1% agarose gel electrophoresis and observed under the UV light. All these qualified PCR products were submitted for sequencing. Species identification was performed based on *CO1* and *16s rRNA* sequences (Fig. S1). In detail, both *CO1* and *16s rRNA* sequence data of each individual were aligned using Clustal W, and the alignments were respectively subjected for phylogenetic tree construction using the maximum likelihood (ML) method. The confidence levels were assessed using the bootstrap procedure with 1000 replications. All these analyses were performed in MEGA 11 software^8^.

### Morphological analysis

After fish were harvested and photographed, the photographs were processed with tpsDIgv.2.12 software to measure the following morphological indicators: total length (L_T_), standard length (L_S_), head length (L_H_), body depth (D_B_), eye diameter (D_E_), depth of caudal peduncle (D_CP_), length of caudal peduncle (L_CP_), length of caudal fin (L_CF_) and height of caudal fin (H_CF_). In addition, six indices, including head length/standard length (L_H_/L_S_), eye diameter/head length (D_E_/L_H_), depth of caudal peduncle/length of caudal peduncle (D_CP_/L_CP_), body depth/standard length (D_B_/L_S_), length of caudal peduncle/standard length (L_CP_/L_S_) and length of caudal fin/length of caudal peduncle (L_CF_/L_CP_), were also used to describe the morphological traits.

To compare the morphological traits of fishes among different populations, cluster analysis was performed for 6 morphological indices to evaluate the differences and distances among populations. This study used morphometric analysis to detect body shape variation and its covariation with other factors. Briefly, after fishes were harvested and photographed, the landmark 2-D Cartesian coordinates were digitized. Eleven landmarks (anatomy feature), including mouth tip, operculum top, first dorsal fin front, first dorsal finback, second dorsal fin front, second dorsal finback, caudal top, caudal bottom, anal finback, anal fin front, and pectoral fin, were selected (Fig. S2), and the coordinates were extracted. Landmark-based geometric morphometric analysis to determine the morphological differences among different populations was carried out by using MorphoJ software^9^.

### Evaluation of population genetic diversity

Standard measures of genetic diversity were evaluated based on *CO1* and *16s rRNA* datasets. The aligned *CO1* and *16s rRNA* sequences were subjected to DnaSP v5^10^ to identify the polymorphic sites and estimate haplotypes data, especially for the number of private haplotypes unique to each population, respectively. The mismatch distribution analysis (MDA) was used to infer the demographic stability of phylogenetic clades and species using DnaSP software. The Tajima’s and Fu’s Ts tests were performed on the Arlequin program^11^. The TCS haplotype network was constructed to estimate the gene genealogies using the statistical parsimony approach at the population level in PopART^12^. Bayesian skyline plot (BSP) was used to examine the historical demographic fluctuation using the BEAST 2.6.0 program^13^.

### Phylogenetic analysis and divergence time estimation

A maximum-likelihood tree was constructed for phylogenetic analyses under the GTR + I + G model in IQtree^14^ based on *CO1* and *16s rRNA* haplotype datasets. The outgroup species used were *Megalobrama amblycephala* and *Scophthalmus maximus* for the *CO1* and *16s rRNA* datasets. As listed in Table S3, the previously reported *CO1* sequences of *E. tetradactylum* and *E. rhadinum*, retrieved from the public NCBI database, were used as the final nucleotide sequence alignment datasets for phylogenetic analyses and divergence time estimation. The target sequences were the haplotypes samples from India, Malaysia, and Vietnam for *CO1* of *E. tetradactylum* and *E. rhadinum*^3,15,16^.

For divergence time estimation, the BEAST analysis based on *CO1* sequences was conducted in BEAST 2.6.0. Program with a lognormal relaxed molecular clock algorithm under the calibrated-Yule model and gamma distribution under the HKY model. Posterior distributions of parameters were estimated using 1,000,000 Markov-chain-Monte Carlo (MCMC) generations sampled every 1000 generations, with a 20% burn-in in the TreeAnnotator 2.6.0 program^17^. The consensus tree was visualized in the FigTree program (https://github.com/rambaut/figtree/releases).

### Ethics declarations

All experimental protocols were approved by the Research Committee of City University of Hong Kong. All methods were carried out in accordance with the relevant guidelines and regulations of the City University of Hong Kong.

## Results and discussion

### Genetic diversity and population structure

**Figure 2:**
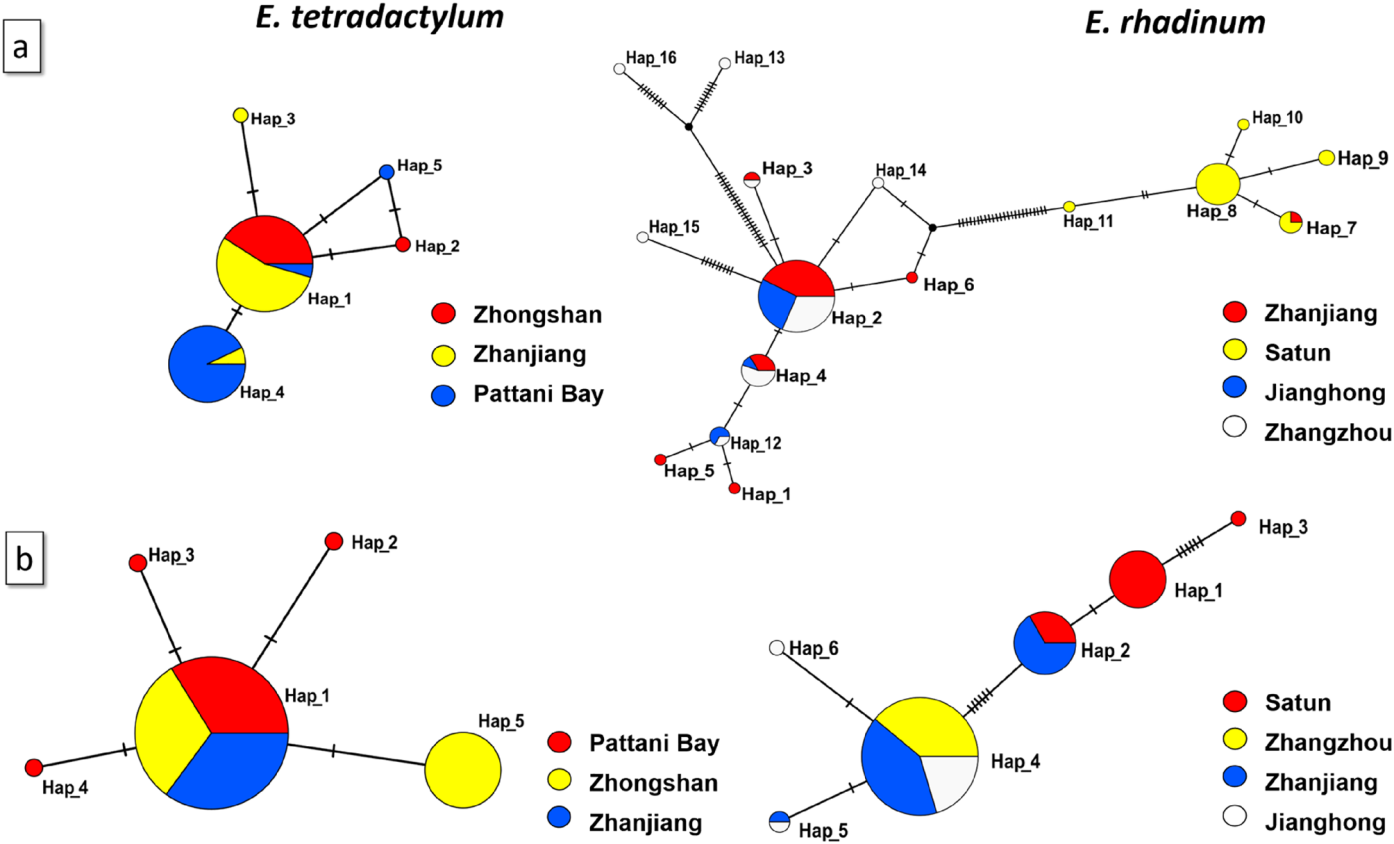
The unrooted TCS haplotype networks were constructed based on the haplotypes of *CO1* (**a**) and *16s rRNA* (**b**) of *Eleutheronema tetradactylum* (left) and *Eleutheronema rhadinum* (right). Haplotype frequency was related to the size of the circle. Different colors within the nodes refer to various sampling sites.

The 614 bp length of mt*CO1* sequences was successfully amplified and sequenced from 75 individuals of *E. tetradactylum* and 89 individuals of *E. rhadinum* from different sites. Based on the *CO1* analysis, we detected 5 and 16 haplotypes, respectively, from *E. tetradactylum* and *E. rhadinum* (Table 1). Only one haplotype was inter-specifically shared in *E. tetradactylum* populations, as showed in the TCS haplotype networks (Fig. 2a). A total of 77 polymorphic sites was identified in *E. rhadinum* but 3 polymorphic sites in *E. tetradactylum*. Among these sites, a total of 3 and 11 parsimoniously informative sites was detected in *E. tetradactylum* and *E. rhadinum*, respectively. In *E. tetradactylum*, the number of *CO1* haplotypes was 2 in ZS and 3 in PA and ZJ. The haplotype diversity was also much higher in ZJ (0.211) and PA (0.197) than ZS (0.105). In *E. rhadinum*, *CO1* haplotypes varied from 3 (JH) to 8 (ZZ). The haplotype diversity was the highest in ZZ (0.663). The populations of ZJ and ZZ showed the statistically negative Tajima’s D value, which could signify the demographic expansion. The MDA revealed similar results (Fig. S3).

**Table 1:** Genetic polymorphisms and neutrality tests of *Eleutheronema tetradactylum* and *Eleutheronema rhadinum* inferred from *CO1* and *16s rRNA*.

Gene	Species	Sites	N	S	Nh	Nph	H	π	Tajima’s D	Fu’s Fs
*CO1*	*E. tetradactylum*	Zhanjiang (ZJ)	27	2	3	1	0.211	0.00035	− 1.233	− 1.543
		Zhongshan (ZS)	19	1	2	1	0.105	0.00017	− 1.165	− 0.838
		Pattani Bay (PA)	29	2	3	1	0.197	0.00043	− 1.009	− 1.168
		Total	75	3	5	3	0.523	0.00091	− 0.675	− 1.388
	*E. rhadinum*	Zhanjiang (ZJ)	27	32	7	3	0.504	0.00449	− 2.477***	0.365
		Jianghong (JH)	15	2	3	0	0.362	0.00096	− 0.105	− 0.124
		Zhangzhou (ZZ)	25	50	8	4	0.663	0.01088	− 2.172**	2.601
		Satun (SA)	22	5	5	4	0.528	0.00113	− 1.471	− 1.957
		Total	89	77	16	11	0.708	0.02127	− 0.762	7.061**
*16 s rRNA*	*E. tetradactylum*	Zhanjiang (ZJ)	27	NA		0	–	–	–	–
		Zhongshan (ZS)	19	NA		1	–	–	–	–
		Pattani Bay (PA)	29	3	4	2	0.200	0.00036	− 1.733	− 3.324
		Total	75	4	5	3	0.442	0.00081	− 0.906	− 1.883
	*E. rhadinum*	Zhanjiang (ZJ)	27	1	2	0	0.074	0.00013	− 1.154	− 1.125
		Jianghong (JH)	15	2	3	1	0.257	0.00047	− 1.490	− 1.546
		Zhangzhou (ZZ)	25	NA	0	0	–	–	–	–
		Satun (SA)	22	7	3	3	0.481	0.00170	− 1.598	1.223
		Total	89	14	6	4	0.454	0.00484	− 0.211	3.483

*Significant at *p* < 0.05. **p < 0.01, ***p < 0.001. N sample size, S number of segregating sites, Nh number of haplotypes, Nph number of private haplotypes, H haplotype diversity, and π nucleotide diversity.

The mitochondrial *16s rRNA* (574 bp in length) was also successfully sequenced from 75 and 89 individuals of *E. tetradactylum* and *E. rhadinum* (Table 1), which yielded 5 and 6 haplotypes, respectively (Fig. 2b). No haplotype was interspecifically shared of *16s rRNA* both in *E. tetradactylum* and *E. rhadinum*. A total of 4 and 14 polymorphic sites of *E. tetradactylum* and *E. rhadinum* were identified, respectively, of which 3 and 4 were parsimoniously informative sites. Table 1 shows that only four haplotypes with 0.200 haplotype diversity were identified in *E. tetradactylum* from PA. In *E. rhadinum*, relatively high haplotype diversity (H = 0.481) and nucleotide diversity (π = 0.00170) were found in populations SA. Overall, the populations from Thailand showed higher genetic diversity than the China population both for *E. tetradactylum* and *E. rhadinum*.

The TCS network was constructed to identify the phylogenetic relationships in *E. tetradactylum* and *E. rhadinum* between China and Thailand populations, as shown in Fig. 2. In *E. tetradactylum*, 5 haplotypes were closely related to a small number of mutation steps, and the Hap_1 was likely the most primitive haplotype, which evolved into others. In *E. rhadinum*, 16 haplotypes were distributed between the two branches, including China and Thailand branches. Only the Hap_7 was shared in ZJ and SA of the Thailand branch. One (hap_1) in *E. tetradactylum* and two (Hap_2 and Hap_8) in *E. rhadinum* were used as the central radiation distribution for most haplotypes. Other haplotypes were formed by a small number of mutations of these haplotypes. As shown in the TCS network of *16s rRNA* haplotypes, the Hap_1 in *E. tetradactylum* and Hap_4 in *E. rhadinum* were the most primitive haplotype, which showed central radiation distributions. Also, in *E. rhadinum*, the haplotypes of China and Thailand populations were divided into two branches; only Hap_2 was shared in ZJ and SA.

The level of population genetic differentiation between China and Thailand populations was also evaluated (Table S3). In *E. tetradactylum*, the average Fixation index (Fst) between PA and the other two sites was 0.81344 in ZS (*p* < 0.05) and 0.73738 in ZJ (*p* < 0.05). In *E. rhadinum*, high value of Fst was observed between SA and other three China population, including ZJ (Fst = 0.93668, *p* < 0.05), JH (Fst = 0.97721, *p* < 0.05) and ZZ (Fst = 0.88497, *p* < 0.05). Overall, pairwise Fst comparisons revealed that *E. tetradactylum* and *E. rhadinum* in China and Thailand were significantly differentiated, indicating low population connectivity among these sites. However, no significant population differentiation was observed within China populations both in *E. tetradactylum* and *E. rhadinum*, which indicated that all China populations did not diverge at the subspecies level. The greater genetic differentiation coefficient means less gene flow among populations. Therefore, due to the gene flow among the China populations, a low level of genetic divergence among different China populations was observed.

Genetic diversity could directly reflect the potential and ability of species to adapt to environmental changes^18,19^. The present study based on mtDNA gene sequences aimed to investigate the genetic diversity of *Eleutheronema* species. The results revealed remarkably low haplotype diversity and nucleotide diversity in all populations. The *E. tetradactylum* showed low levels of variability, which might reflect the impact of human activities, including marine pollution and overfishing^20^. Although *E. rhadinum* showed relatively higher genetic diversity than *E. tetradactylum*, its genetic diversity levels were still remarkably lower than other marine organisms distributed in the China coastal water^21–23^. Since *E. tetradactylum* was classified as endangered by the IUCN, more attention should be paid to conserve the imperiled *E. tetradactylum* and *E. rhadinum*. Additionally, the low genetic diversity of ZS strongly indicated the necessities to strengthen the genetic management of artificial breeding populations to ensure that artificial breeding populations have a higher level of genetic diversity. A low level of genetic divergence among different populations in China suggested genetic similarities in the Chinese coastal water. Generally, genetic homogeneity in marine fishes can be attributed to the absence of barriers between ocean basins and adjacent continental margins. The ocean currents in the China sea could facilitate the dispersal of marine larvae, and may be responsible for the genetic homogeneity. A previous study indicated that the dispersal of *E. tetradactylum* was sufficiently low, which had lower swimming performance and poor orientation and can be effortlessly hindered by the geographical barrier^24^. Thus, limited communication between China and Thailand populations caused the high genetic differentiation in all groups. Our results implied that the limited ecological population connectivity of local China populations might permit self-recruitment rather than passive dispersal. Therefore, low genetic diversity and shallow population structure of *E. tetradactylum* and *E. rhadinum* resulted in a serious concern about fisheries management and conservation of these two *Eleutheronema* species.

### Reconstruction of CO1 phylogenetic relationships and demography

**Figure 3:**
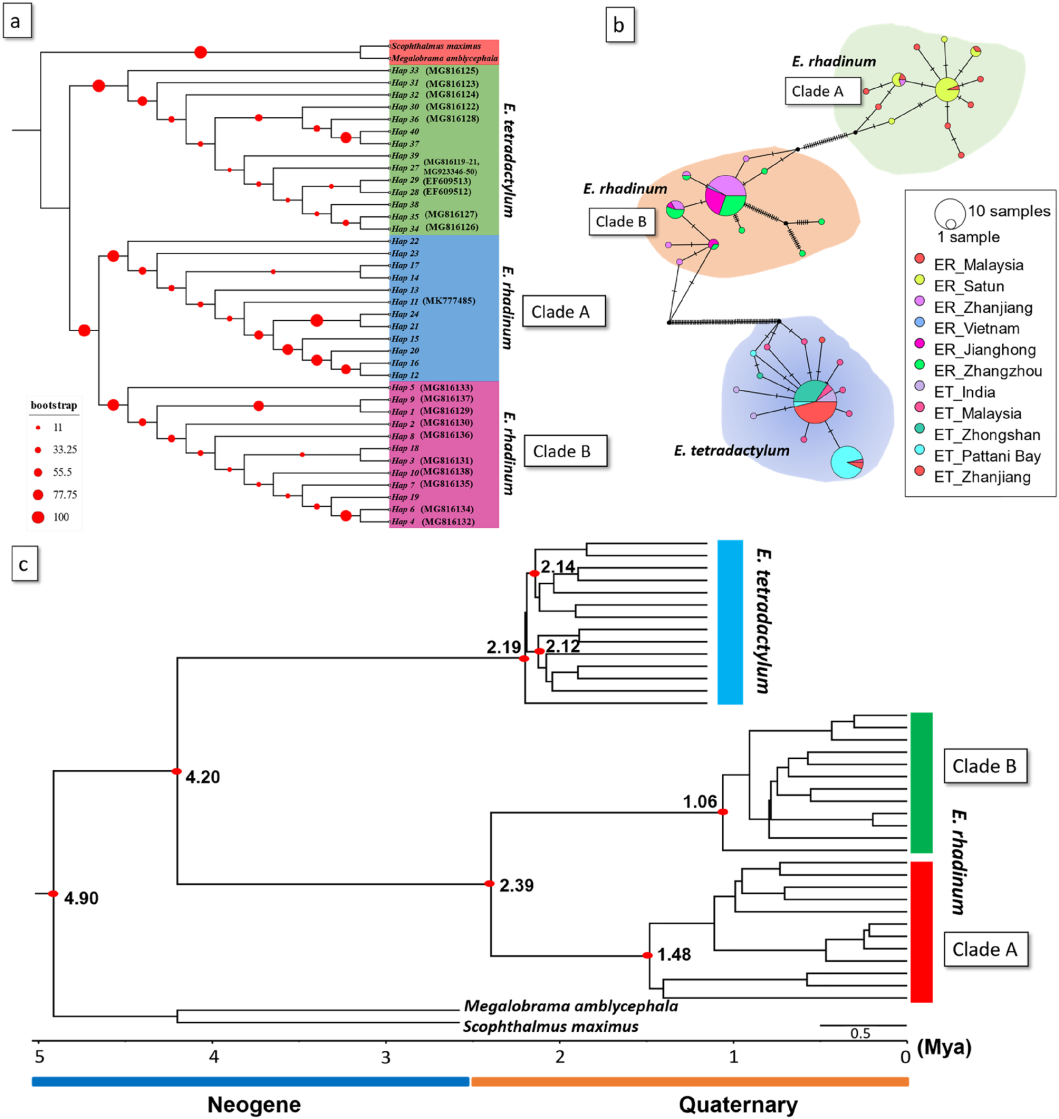
(**a**) Phylogenetic tree reconstructed based on the maximum likelihood (ML) methods using *CO1* haplotypes of *Eleutheronema tetradactylum* and *Eleutheronema rhadinum*. Two species, including *Megalobrama amblycephala* and *Scophthalmus maximus*, were used as outgroups. (**b**) The unrooted TCS haplotype networks were constructed based on the haplotypes of *CO1* from each population of *E. tetradactylum* and *E. rhadinum*. Haplotype frequency was related to the size of the circle. Different colors within the nodes refer to various sampling sites. (**c**) Time-calibrated Bayesian tree reconstructed with 13 and 24 *CO1* haplotypes of *E. tetradactylum* and *E. rhadinum*, respectively, using the BEAST program to infer ancestral areas under the Bayesian binary MCMC (BBM) model.

To determine the phylogenetic relationships of *E. tetradactylum* and *E. rhadinum* in China and Thailand with those elsewhere in the West-Pacific region, we used our newly generated *CO1* sequences and publicly available *CO1* DNA sequences from NCBI’s GenBank database. We retrieved 9 haplotypes from 17 sequences for *E. tetradactylum* and 8 haplotypes from 12 sequences for *E. rhadinum* from the public database (Table 2, Table S4). Two haplotypes for two outgroup species were also retrieved. With 5 haplotypes for *E. tetradactylum* and 16 haplotypes for *E. rhadinum* newly obtained in this study, 14 and 24 haplotypes of *E. tetradactylum* and *E. rhadinum* were obtained, respectively, and used for the phylogenetic and population genetic analyses. According to the ML phylogenetic trees (Fig. 3a), *E. tetradactylum* and *E. rhadinum* formed strong independent monophyletic groups with high node confidence values and were clearly separated by outgroup species. Within the group of *E. rhadinum*, there were two genetic lineages of the Clade A and B. For *E. tetradactylum*, however, only single clade was formed irrespective of their geographic affinity, which was similar to *Pethia conchonius* population pattern from India as a result of slow evolutionary rate in this specie or the occurrence of incomplete lineage shorting in *CO1* gene^25^. In the TCS network analyses (Fig. 3b), *E. tetradactylum* was evidently separated from *E. rhadinum*, as shown in the phylogenetic analyses. For *E. rhadinum*, the Clade A consisted of China (ZJ), Malaysia, and Thailand (Satun province). On the other hand, the haplotypes of Clade B were from China (ZZ, JH, and ZJ) and Vietnam. Thus, *CO1* haplotypes of *E. rhadinum* collected from China and Thailand could be allocated into Clade A and B. Haplotypes from the Clade A and B coexisted only in population ZJ. Additionally, a star-like pattern appeared in the haplotype network in Clade B of *E. rhadinum*, suggesting the signature of demographic expansion in the process of dispersal. Moreover, some low frequency haplotypes were also identified in Clade B, that may have originated as a result of adaptation to the conditions in this area^26^. However, these populations of *E. tetradactylum* did not go through recent population expansion as star-like topology was the result of population expansion^27^.

**Table 2:** Neutrality tests with *CO1* for *Eleutheronema tetradactylum* and *Eleutheronema rhadinum*.

Species	Haplotype no	Clade	Tajima’s D	Fu’s Fs
*E. tetradactylum*	14	–	− 1.917*	− 11.37
*E. rhadinum*	12	Clade A	− 1.943*	− 8.889
	12	Clade B	− 2.581***	− 0.605
	24	Clade A + B	− 0.469	3.728*

*Significant at p < 0.05. **p < 0.01, ***p < 0.001.

**Figure 4:**
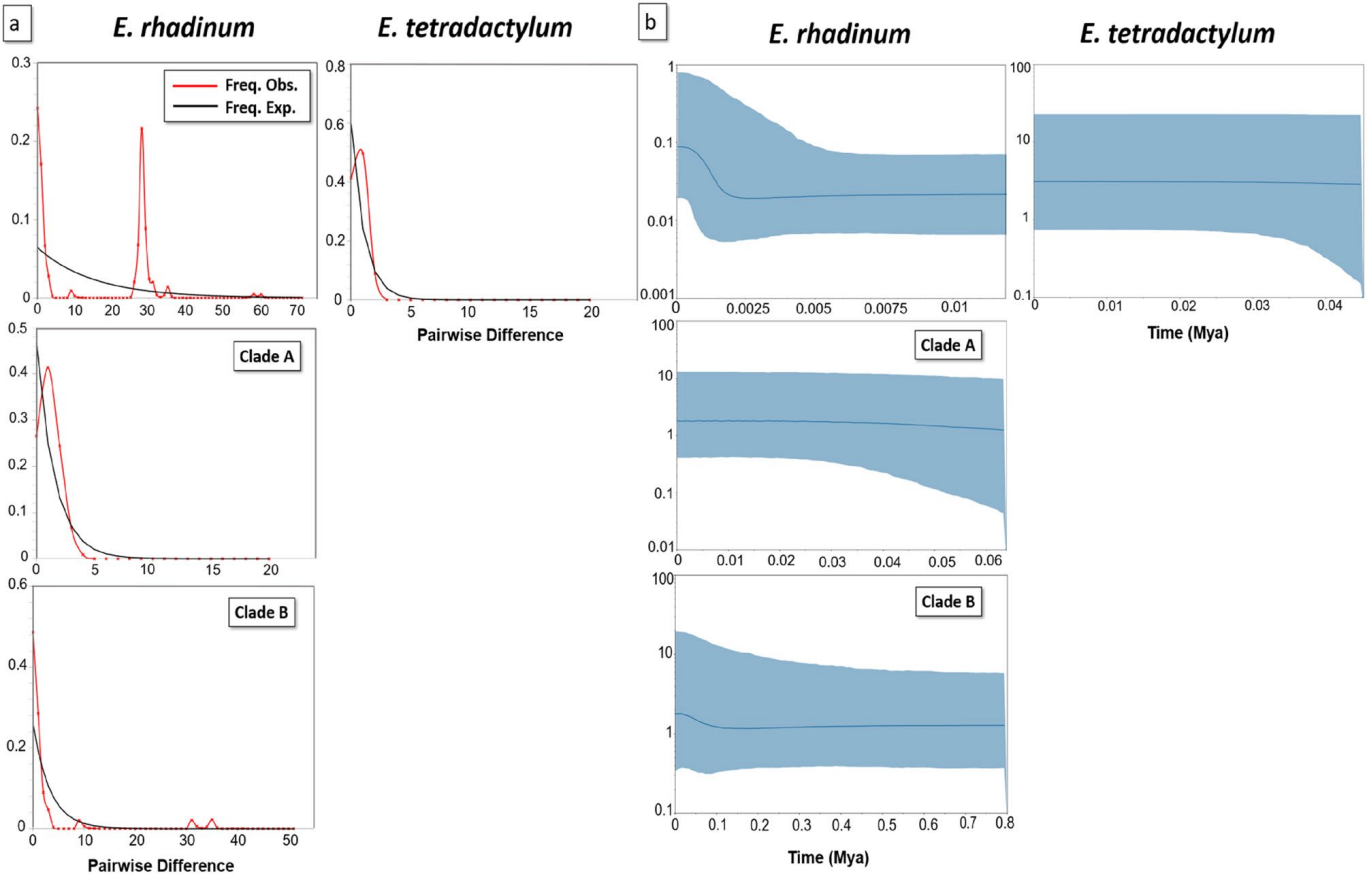
(**a**) The mismatch distribution analysis (MDA) and (**b**) Bayesian skyline plot (BSP) estimated based on the *CO1* haplotypes of *Eleutheronema rhadinum* Clades of A and B and *Eleutheronema tetradactylum*, respectively. The number of haplotypes for the clades of *E. tetradactylum* and *E. rhadinum* refers to Table S4.

The neutrality test was performed with 14 and 24 *CO1* haplotypes of *E. tetradactylum* and *E. rhadinum*, respectively. All these clades in the *CO1* data showed negative values in Tajima’s D and Fu’s Fs, but only the Tajima’s D values were significant. By testing the species itself, the same pattern disappeared, and the value of Fu’s Fs was only significant in *E. rhadinum*. Based on the mismatch distribution analyses (MDA) on *CO1* for each species (Fig. 4a), *E. rhadinum* showed multi-modal curves, indicating the probability that two slightly different genetic groups existed within *E. rhadinum*. In addition, Clade B showed unimodal curves in *E. rhadinum*, indicating the probability of genetic structure within Clade B, but not in Clade A, despite a definite genetic structure within the whole *E. rhadinum* clades. In *E. tetradactylum*, the mismatch distribution of all populations was not typically unimodal, which indicated no population selection or expansion in these populations, consistent with the results from mitochondrial DNA Cytb sequence^6^. Totally, the MDA analysis based on *CO1* of *E. rhadinum* showed a more complicated multi-modal curve than that of *E. tetradactylum*.

Bayesian skyline plot (BSP) analyses based on *CO1* haplotypes were used to test the fluctuation pattern in effective population size of *E. tetradactylum* and *E. rhadinum*, including *E. rhadinum* clades of A and B (Fig. 4b). In *E. rhadinum*, the effective population size increased slightly from 0.0003 Mya but was ceased at around 0.0020 Mya. Among the two clades within *E. rhadinum*, slight growth events were only observed in Clade B at approximately 0.02 Mya. The molecular clock analysis estimated that *E. tetradactylum* and *E. rhadinum* shared a common ancestor, about 4.20 Mya. The estimated divergence time of *E. rhadinum* was around 2.39 Mya. Within *E. rhadinum*, Clade A first diverged off about 1.48 Mya, and the Clade B diverged at approximately 1.06 Mya (Fig. 3c).

Overall, the newly obtained and previously reported data were applied to estimate the phylogenetic relationships and demographic analysis, which provided strong evidence for a shared common origin or ancestor of *E. tetradactylum and E. rhadinum*, since *E. rhadinum* had long been recognized as a junior synonym of *E. tetradactylum*^1,28^. Moreover, the results indicated the probability that two slightly different genetic groups existed within *E. rhadinum* due to genetic breakdown, including Clade A and B. Similar patterns were also observed in the population of *Sardina pilchardus*, and the causes for the isolation of the population may be related to oceanographic barriers^29^. Individuals belonging to Clade A were dominantly distributed in Malaysia and Thailand and may not have the opportunity of demographic expansion on the Malay Peninsula. Also, previous study indicated that Malay Peninsula played a role in shaping the contemporary genetic structure among populations of *Pampus chinensis*^30^. However, demographic expansion occurred in Clade B, which mainly included China populations and was consistent with previous population structure analysis that *E. rhadinum* in the East and South China Seas developed divergent genetic structures and experienced a population expansion^5^. To better understand the population dynamics of *Eleutheronema* species, whole genome resequencing analysis needs to be conducted in further population genetic studies of adaptation and natural selection of *Eleutheronema* species.

### Morphological analysis

**Figure 5:**
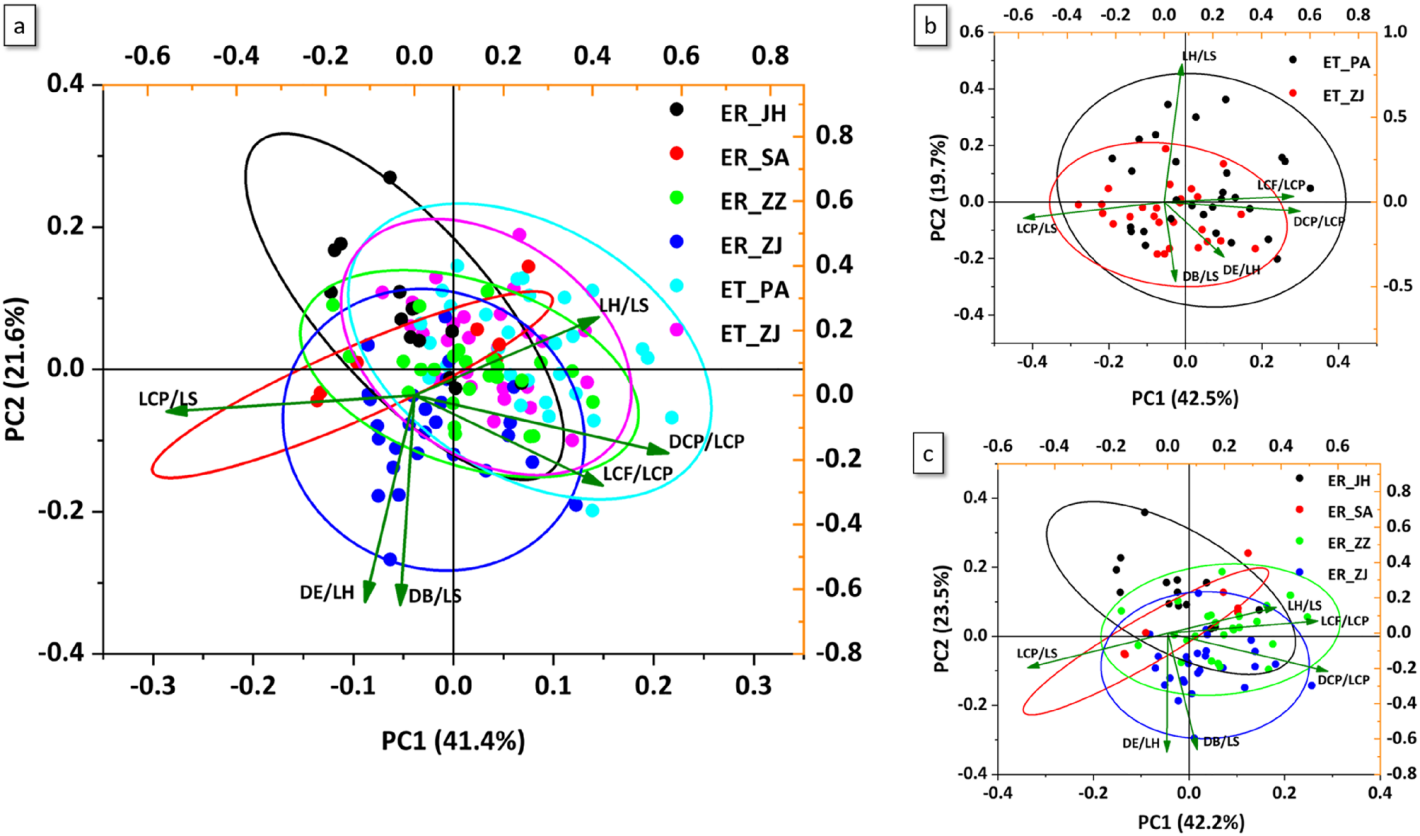
Principal component analysis depicting the first two axes of morphological variation of both *Eleutheronema tetradactylum* and *Eleutheronema rhadinum* populations (**a**), the *E. tetradactylum* (**b**), and *E. rhadinum* (**c**), performed in R.

In this study, principal component analysis (PCA) based on 6 morphometric variables among six populations showed certain degrees of overlap, and PC1 and PC2 showed 41.4% and 21.6% of the total variance, respectively (Fig. 5a, Tables S5 and S6). Thus, PC1 was the most crucial component contributing to separation among populations. In *E. tetradactylum* and *E. rhadinum*, the PCA of all 6 morphometric variables extracted 3 principal components (PC1, PC2, and PC3), explaining 81.27% and 81.05%of the total variance, respectively (Table S7, Fig. 5b, c). The PC1 contributed the highest variance of the total variability in *E. tetradactylum* and *E. rhadinum*, which accounted for 42%. In *E. tetradactylum*, the component matrix of PCA revealed that the L_CP_/L_S_, D_CP_/L_CP_, and L_CF_/L_CP_ of 3 variables were relatively high loadings on PC1 (Table S6). However, variations in PC2 and PC3 were primarily contributed from the L_H_/L_S_ (− 0.81379) and D_B_/L_S_ (0.72021). In *E. rhadinum*, the 2 variables with high loadings on PC1 were D_CP_/L_CP_ (0.56505) and L_CF_/L_CP_ (0.52844). Strong loading of characters involved in D_E_/L_H_ (0.67347) and L_H_/L_S_ (0.69152) was also respectively observed in the case of PC2 and PC3.

**Figure 6:**
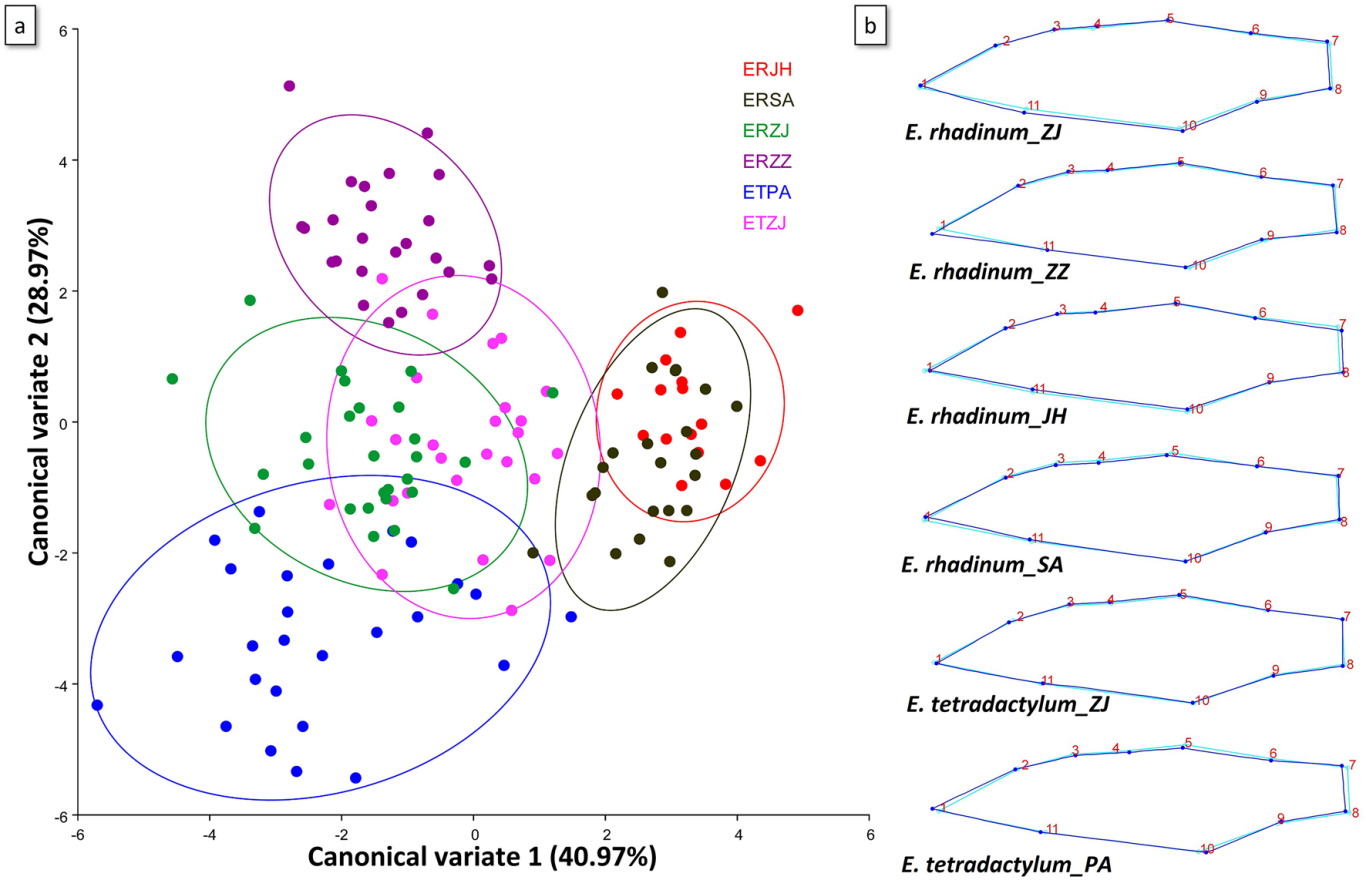
Landmark-based geometric morphometric analysis of *E. tetradactylum* and *E. rhadinum*. (**a**) Canonical variate analyses; (**b**) Shape change.

Subsequently, CAV based on the *p* value of the permutation test was used to describe the shape variations among populations. Results of CVA and grouping of 6 populations in the two canonical variables for each species are shown in Fig. 6. Obviously, the studied populations occupied different areas. The scatter plot from CV1 (40.97%) and CV2 (28.97%) showed that the body shape of *E. tetradactylum* and *E. rhadinum* were clearly separated into distinct clusters in PA and ZZ. *E. rhadinum* from both geographical regions (JH and SA) was not clearly isolated along the first two canonical variate axes. Moreover, *E. tetradactylum* and *E. rhadinum* from ZJ also showed certain degrees of overlap (Fig. 6). Mahalanobis and Procrustes distances (Table S8) by pairwise comparisons among populations showed significant differences (*p* < 0.0001).

To adapt to changeable environments, fish modify their morphology and physiology, and the phenotypic plasticity results in morphological divergence which may, in some instances, be involved in response to different environmental conditions^31^. Our study revealed the phenotypic divergence of *Eleutheronema* species among diverse populations, characterized mainly by the depth of caudal peduncle and length of caudal peduncle, indicating the evolution in the caudal peduncle which is associated with swimming behaviors in deeper waters^32^. The caudal fin was also among the variables with high loadings on the PC1, suggesting evolution in the caudal area, likely associated with the consequence of phenotypic plasticity in response to hydrological conditions. A high morphological divergence was also reported in *Eleutheronema* collected from China, suggesting rapid and apparently adaptive morphological divergence of *Eleutheronema* species in response to changes in China coastal water^33^. However, some morphometric and meristic characteristics were not distinct between *E. tetradactylum* and *E. rhadinum* in Zhanjiang (ZJ), suggesting that *E. tetradactylum* and *E. rhadinum* shared a common ancestor, and the phenotypic modifications might be mainly due to the adaptation to local habitats of Zhanjiang. The morphological analysis also revealed that different local environmental conditions of China and Thailand coastal water might have influenced the *Eleutheronema* differently, as evidenced by morphological modifications to better adapt and survive in the local ecosystem. Overall, the morphological divergence among the different populations in China and Thailand coastal water reflected the geographic isolation underlying the population structure and specific local adaptations to environmental changes.

## Conclusion

The present genetic and morphological data provided novel genetic information on the population genetic structure and demographic history of *Eleutheronema* species in China and Thailand coastal waters. Low genetic diversity and shallow population structure were observed in *E. tetradactylum* and *E. rhadinum*, which suggested the need for considerable fisheries management and conservation. The phylogenetic relationships and population genetic structures provided strong evidence for a shared common origin or ancestor of *E. tetradactylum* and *E. rhadinum*. In Indo-West-Pacific regions, the *E. rhadinum* populations were likely subdivided into two genetic lineages, including Clade A and B. The Thailand and Malaysia populations, which belonged to Clade A, may have no demographic opportunity during dispersing. However, demographic expansion occurred slightly in Clade B, mainly distributing in the China's coastal water. Our present study provided important understanding of the genetics and morphology of *E. tetradactylum* and *E. rhadinum* and have implications for future conservation and management efforts for *Eleutheronema* species.

## Supplementary Information

Supplementary Information.
