# Supplementary material for: Molecular phylogenetic and morphometric analysis of population structure and demography of endangered threadfin fish Eleutheronema from Indo-Pacific waters
Source: Sci Rep. 2022 Mar 2;12:3455. doi: 10.1038/s41598-022-07342-w (PMC8891298; doi:10.1038/s41598-022-07342-w)
Supplement: Supplementary file 1 — Supplementary Information. [file 41598_2022_7342_MOESM1_ESM.docx]

**Supporting Information for**

**Molecular phylogenetic and morphometric analysis of population structure and demography of endangered threadfin fish** ***Eleutheronema* from Indo-Pacific waters**

Jie Xiao^1^, Shaoliang Lyu^1^, Teuku H. Iqbal^2^, [Sukree Hajisamae](https://onlinelibrary.wiley.com/action/doSearch?ContribAuthorRaw=Hajisamae%2C+Sukree)^2^, Karl W.K. Tsim^3^,

Wen-Xiong Wang^1,4^*

*^1^School of Energy and Environment and State Key Laboratory of Marine Pollution, City University of Hong Kong, Kowloon, Hong Kong, China*

*^2^Faculty of Science and Technology, Prince of Songkla University, Pattani 94000, Thailand.*

*^3^Division of Life Science, Hong Kong University of Science and Technology, Clear Water Bay, Kowloon, Hong Kong, China*

*^4^Research Centre for the Oceans and Human Health, City University of Hong Kong Shenzhen Research Institute, Shenzhen 518057, China*

*Corresponding author, Email: wx.wang@cityu.edu.hk

**Figure S1.** Phylogenetic tree constructed using 164 *CO1* (a) and 164 *16s rRNA* (b) sequences of *Eleutheronema tetradactylum* and *Eleutheronema rhadinum* based on maximum likelihood (ML) methods


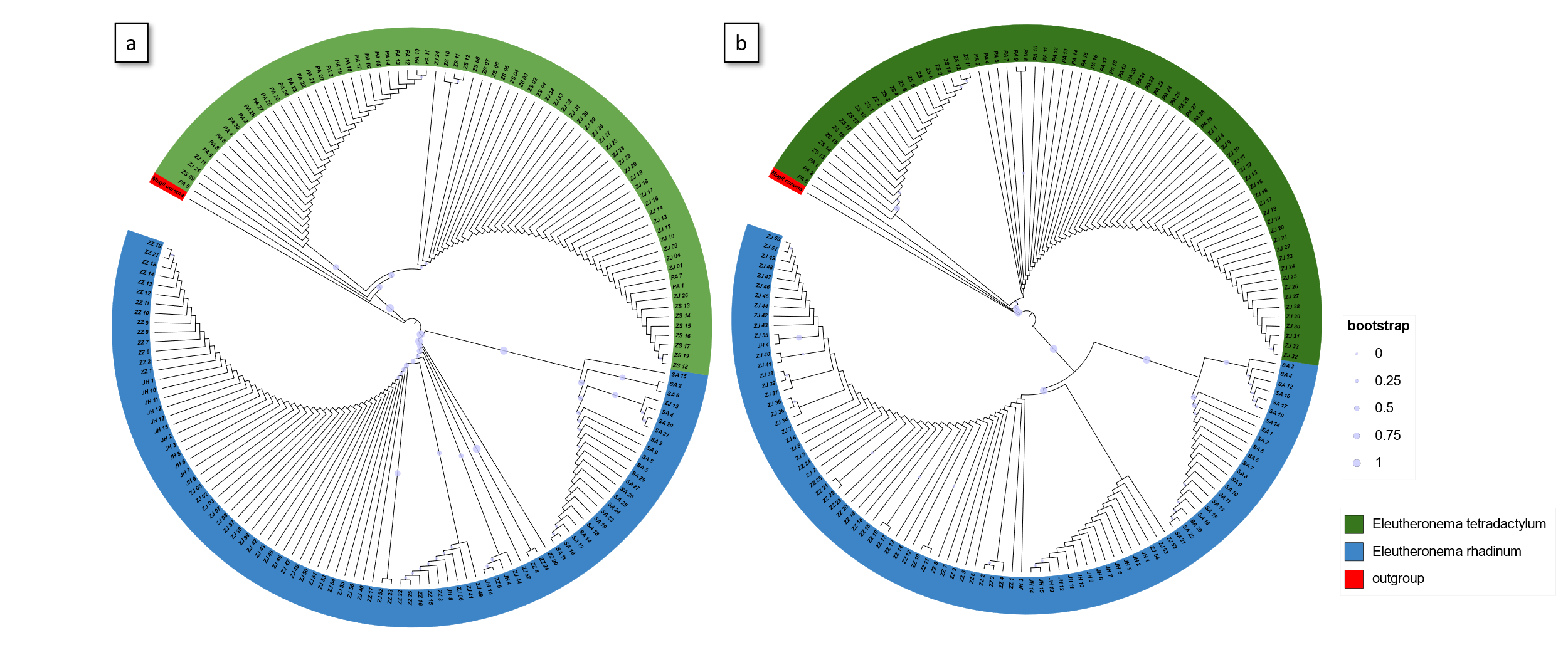


**Fig S2**. Landmarks coordinates transformation. 1, mouth tip. 2, operculum top. 3, the first dorsal fin front. 4, the first dorsal fin back. 5, the second dorsal fin front. 6, the second dorsal fin back. 7, caudal top. 8, caudal bottom. 9, anal fin back. 10, anal fin front. 11, pectoral fin.


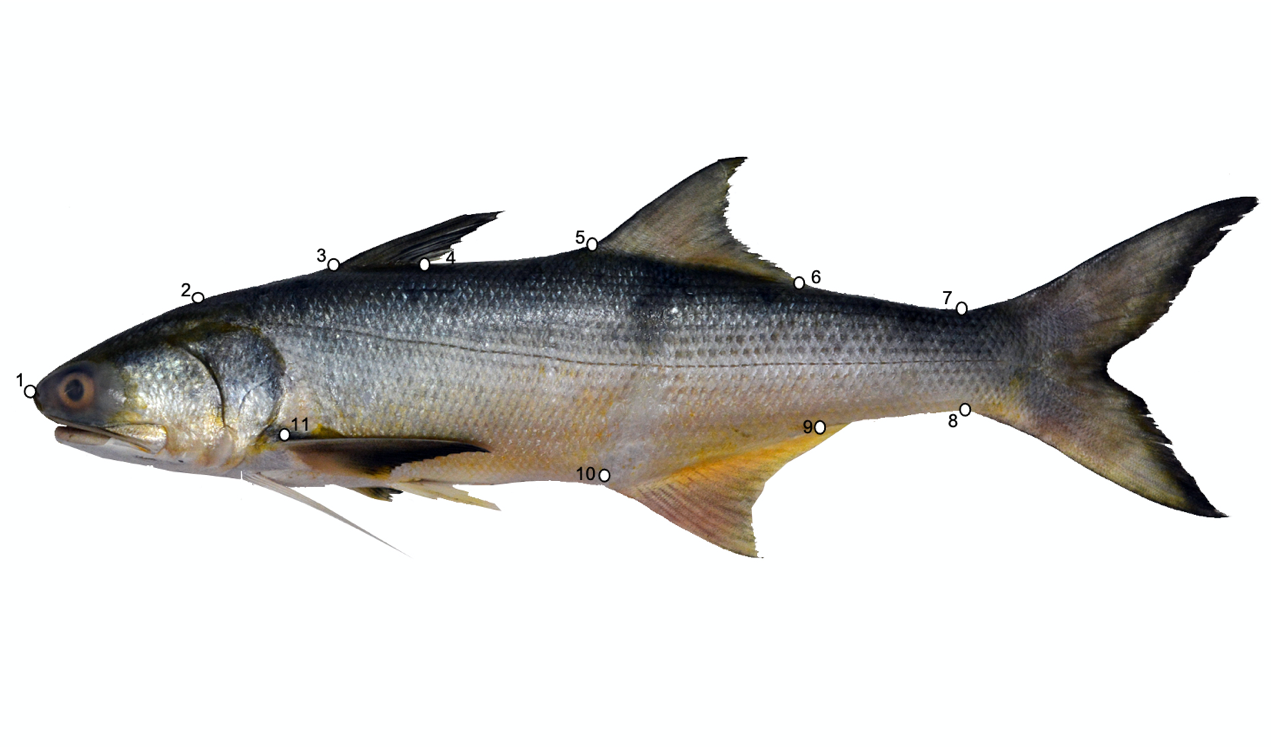


**Figure S3**. The mismatch distribution analysis (MDA) estimated based on the *CO1* haplotypes of (a) *Eleutheronema tetradactylum* and (b) *Eleutheronema rhadinum*


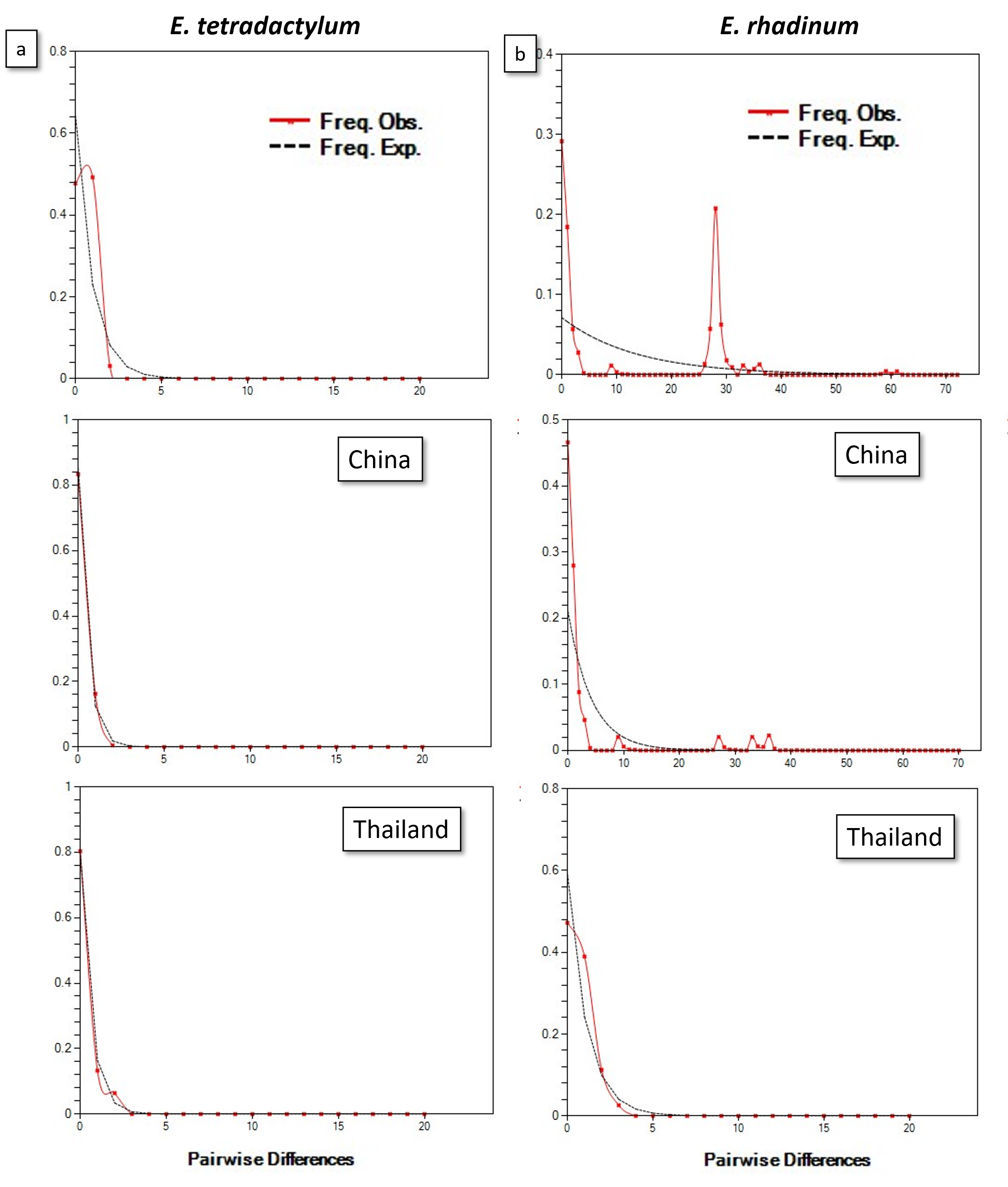


**Table S1**. Population information and geographical characteristics of *E. tetradactylum* and *E. rhadinum.*

| **Population** | **N** | **Location** | **Latitude** | **Longitude** | **Year** |
| --- | --- | --- | --- | --- | --- |
| **Zhanjiang (ZJ)** | 54 | China | 20.70 | 110.55 | 2020/10-11 |
| **Jianghong (JH)** | 15 | China | 21.05 | 109.45 | 2020/11-12 |
| **Zhangzhou (ZZ)** | 25 | China | 24.25 | 118.08 | 2021/3-4 |
| **Zhongshan (ZS)** | 19 | China | 22.51 | 113.39 | 2021/6 |
| **Pattani Bay (PA)** | 29 | Thailand | 6.544 | 101.17 | 2020/11 |
| **Satun (SA)** | 22 | Thailand | 6.291 | 100.04 | 2020/12 |

**Table S2**. *CO1* sequences of *E. tetradactylum* and *E. rhadinum* used in this study.

| **Species** | **Location** | **Accession number** | **Reference** |
| --- | --- | --- | --- |
| ***E. tetradactylum*** | Malaysia | MG816119-28 | (Atikah et al. 2020) |
|  | India | EF609512-13, MG923346-50 | (Lakra et al. 2011) |
| ***E. rhadinum*** | Malaysia | MG816129-38 | (Atikah et al. 2020) |
|  | Vietnam | MK777485 | (Thu et al. 2019) |

**Table S3**. Pairwise Fst values for *CO1* sequences in *E. tetradactylum* and *E. rhadinum.*

|  | **Location** | **Zhongshan** | **Zhanjiang** |  |
| --- | --- | --- | --- | --- |
| ***E. tetradactylum*** | **Zhongshan** |  |  |  |
|  | **Zhanjiang** | 0.01740 |  |  |
|  | **Pattani bay** | **0.81344** | **0.73738** |  |
|  |  | **Zhanjiang** | **Satun** | **Jianghong** |
|  | **Zhanjiang** |  |  |  |
| ***E. rhadinum*** | **Satun** | **0.93668** |  |  |
|  | **Jianghong** | -0.01308 | **0.97721** |  |
|  | **Zhangzhou** | 0.01430 | **0.88497** | 0.02019 |

**Table S4**. Haplotypes of *CO1* sequences identified in *E. tetradactylum* and *E. rhadinum* populations.

|  | *E. rhadinum* | | | | | | *E. tetradactylum* | | | | |
| --- | --- | --- | --- | --- | --- | --- | --- | --- | --- | --- | --- |
|  | Malaysia | SA | ZJ | Vietnam | JH | ZZ | India | Malaysia | ZS | PA | ZJ |
| Hap_1 | 1 | 0 | 0 | 0 | 0 | 0 | 0 | 0 | 0 | 0 | 0 |
| Hap_2 | 1 | 0 | 0 | 0 | 0 | 0 | 0 | 0 | 0 | 0 | 0 |
| Hap_3 | 1 | 0 | 0 | 0 | 0 | 0 | 0 | 0 | 0 | 0 | 0 |
| Hap_4 | 1 | 0 | 0 | 0 | 0 | 0 | 0 | 0 | 0 | 0 | 0 |
| Hap_5 | 1 | 0 | 0 | 0 | 0 | 0 | 0 | 0 | 0 | 0 | 0 |
| Hap_6 | 1 | 0 | 0 | 0 | 0 | 0 | 0 | 0 | 0 | 0 | 0 |
| Hap_7 | 1 | 2 | 0 | 0 | 0 | 0 | 0 | 0 | 0 | 0 | 0 |
| Hap_8 | 1 | 0 | 0 | 0 | 0 | 0 | 0 | 0 | 0 | 0 | 0 |
| Hap_9 | 1 | 3 | 1 | 0 | 0 | 0 | 0 | 0 | 0 | 0 | 0 |
| Hap_10 | 1 | 15 | 0 | 0 | 0 | 0 | 0 | 0 | 0 | 0 | 0 |
| Hap_11 | 0 | 0 | 0 | 1 | 0 | 0 | 0 | 0 | 0 | 0 | 0 |
| Hap_12 | 0 | 0 | 1 | 0 | 0 | 0 | 0 | 0 | 0 | 0 | 0 |
| **Hap_13** | 0 | 0 | 19 | 0 | 12 | 14 | 0 | 0 | 0 | 0 | 0 |
| Hap_14 | 0 | 0 | 1 | 0 | 0 | 1 | 0 | 0 | 0 | 0 | 0 |
| Hap_15 | 0 | 0 | 3 | 0 | 1 | 5 | 0 | 0 | 0 | 0 | 0 |
| Hap_16 | 0 | 0 | 1 | 0 | 0 | 0 | 0 | 0 | 0 | 0 | 0 |
| Hap_17 | 0 | 0 | 1 | 0 | 0 | 0 | 0 | 0 | 0 | 0 | 0 |
| Hap_18 | 0 | 1 | 0 | 0 | 0 | 0 | 0 | 0 | 0 | 0 | 0 |
| Hap_19 | 0 | 1 | 0 | 0 | 0 | 0 | 0 | 0 | 0 | 0 | 0 |
| Hap_20 | 0 | 0 | 0 | 0 | 2 | 1 | 0 | 0 | 0 | 0 | 0 |
| Hap_21 | 0 | 0 | 0 | 0 | 0 | 1 | 0 | 0 | 0 | 0 | 0 |
| Hap_22 | 0 | 0 | 0 | 0 | 0 | 1 | 0 | 0 | 0 | 0 | 0 |
| Hap_23 | 0 | 0 | 0 | 0 | 0 | 1 | 0 | 0 | 0 | 0 | 0 |
| Hap_24 | 0 | 0 | 0 | 0 | 0 | 1 | 0 | 0 | 0 | 0 | 0 |
| **Hap_27** | 0 | 0 | 0 | 0 | 0 | 0 | 5 | 3 | 18 | 2 | 23 |
| Hap_28 | 0 | 0 | 0 | 0 | 0 | 0 | 1 | 0 | 0 | 0 | 0 |
| Hap_29 | 0 | 0 | 0 | 0 | 0 | 0 | 1 | 0 | 0 | 0 | 0 |
| Hap_30 | 0 | 0 | 0 | 0 | 0 | 0 | 0 | 1 | 0 | 0 | 0 |
| Hap_31 | 0 | 0 | 0 | 0 | 0 | 0 | 0 | 1 | 0 | 0 | 0 |
| Hap_32 | 0 | 0 | 0 | 0 | 0 | 0 | 0 | 1 | 0 | 0 | 0 |
| Hap_33 | 0 | 0 | 0 | 0 | 0 | 0 | 0 | 1 | 0 | 0 | 0 |
| Hap_34 | 0 | 0 | 0 | 0 | 0 | 0 | 0 | 1 | 0 | 0 | 0 |
| Hap_35 | 0 | 0 | 0 | 0 | 0 | 0 | 0 | 1 | 0 | 0 | 0 |
| **Hap_36** | 0 | 0 | 0 | 0 | 0 | 0 | 0 | 1 | 0 | 26 | 2 |
| Hap_37 | 0 | 0 | 0 | 0 | 0 | 0 | 0 | 0 | 1 | 0 | 0 |
| Hap_38 | 0 | 0 | 0 | 0 | 0 | 0 | 0 | 0 | 0 | 0 | 1 |
| Hap_39 | 0 | 0 | 0 | 0 | 0 | 0 | 0 | 0 | 0 | 0 | 1 |
| Hap_40 | 0 | 0 | 0 | 0 | 0 | 0 | 0 | 0 | 0 | 1 | 0 |

Hap_25 and Hap_26 represent the haplotype of outgroup, *Megalobrama amblycephala* and *Scophthalmus maximus*

**Table S5-1**. Phenotypic variation within and among the populations of *Eleutheronema tetradactylum* concerning morphometric variables, unit: cm.

|  | **Sites** |  |  |  |
| --- | --- | --- | --- | --- |
|  | **Zhanjiang, n= 28** | | **Pattani Bay, n= 30** | |
| ***L_T_*** | 45.54 ± 4.09 | (37.83-51.97) | 36.98 ± 2.16 | (31.29-41.14) |
| ***L_S_*** | 37.38 ± 3.43 | (31.31 -42.59) | 30.2 ± 1.79 | (25.04-33.85) |
| ***L_H_*** | 9.48 ± 0.89 | (7.85-11.07) | 8.04 ± 1.7 | (6.10-9.23) |
| ***D_B_*** | 9.23 ± 0.87 | (7.90-11.04) | 7.35 ± 0.5 | (5.92-8.30) |
| ***D_E_*** | 1.59 ± 0.20 | (1.24-1.96) | 1.45 ± 0.16 | (1.07-1.72) |
| ***D_CP_*** | 3.99 ± 0.37 | (3.30-4.47) | 3.19 ± 0.27 | (2.61-3.80) |
| ***L_CP_*** | 5.87 ± 0.64 | (4.74-6.95) | 4.54 ± 0.32 | (3.96-5.25) |
| ***L_CF_*** | 9.83 ± 0.90 | (8.26-11.15) | 7.87 ± 0.78 | (6.24-9.77) |
| ***H_CF_*** | 11.13 ± 1.62 | (7.40-13.94) | 7.08 ± 0.94 | (4.89-9.71) |

***L_T_***: total Length; ***L_S_***: standard Length; ***L_H_***: head Length; ***D_B_***: body depth; ***D_E_***: eye diameter; ***D_CP_***: depth of caudal peduncle; ***L_CP_***: Length of caudal peduncle; ***L_CF_***: Length of caudal fin; ***H_CF_***: height of caudal fin.

**Table S5-2**. Phenotypic variation within and among the populations of *Eleutheronema rhadinum* concerning morphometric variables, unit: cm.

|  | **Sites** |  |  |  |  |  |  |  |
| --- | --- | --- | --- | --- | --- | --- | --- | --- |
|  | **Zhanjiang, n= 28** | | **Jianghong, n= 16** | | **Satun, n=28** | | **Zhangzhou, n=25** | |
| ***L_T_*** | 32.02 ±3.97 | (27.43-49.24) | 55.20 ±3.23 | (49.66-59.87) | 49.71±5.47 | (31.96-62.03) | 24.41±4.21 | (19.10-31.93) |
| ***L_S_*** | 25.45 ±3.09 | (21.66 -38.80) | 44.55 ±2.82 | (40.29-49.52) | 40.02±4.29 | (25.59-49.21) | 19.10±3.25 | (14.95-25.12) |
| ***L_H_*** | 6.19 ± 0.83 | (5.13-9.60) | 10.39 ± 0.72 | (9.11-11.60) | 9.24±1.11 | (6.45-13.19) | 5.12±0.82 | (3.96-6.48) |
| ***D_B_*** | 6.48 ± 0.78 | (5.77-9.79) | 10.71 ± 0.82 | (9.62-12.19) | 9.83±1.16 | (5.83-12.49) | 4.65±0.79 | (3.52-6.11) |
| ***D_E_*** | 1.20 ±0.12 | (0.96-1.57) | 1.57 ± 0.23 | (1.24-2.08) | 1.77±0.18 | (1.19-2.06) | 0.98±0.13 | (0.73-1.27) |
| ***D_CP_*** | 2.77 ± 0.33 | (2.36-4.16) | 4.13 ± 0.29 | (3.59-4.69) | 3.89±0.39 | (2.46-4.81) | 2.08±0.34 | (1.52-2.66) |
| ***L_CP_*** | 4.32 ±0.66 | (3.46-7.03) | 7.39 ± 0.65 | (6.53-8.56) | 6.98±1.05 | (3.59-7.87) | 3.29±0.65 | (2.39-4.45) |
| ***L_CF_*** | 7.47 ± 0.81 | (6.24-10.56) | 12.53 ± 0.81 | (11.29-13.91) | 11.64±1.53 | (7.30-14.88) | 5.99±1.04 | (4.46-7.72) |
| ***H_CF_*** | 7.09 ± 1.37 | (4.91-10.53) | 11.70 ± 1.26 | (9.25-13.60) | 11.29±2.05 | (6.12- 16.47) | 6.34±1.17 | (4.53-8.86) |

***L_T_***: total Length; ***L_S_***: standard Length; ***L_H_***: head Length; ***D_B_***: body depth; ***D_E_***: eye diameter; ***D_CP_***: depth of caudal peduncle; ***L_CP_***: Length of caudal peduncle; ***L_CF_***: Length of caudal fin; ***H_CF_***: height of caudal fin.

**Table S6**. Six morphological traits of *E. tetradactylum* and *E. rhadinum.*

| **Species** | **Location** | **L_H_/L_S_** | **D_E_/L_H_** | **D_CP_/L_CP_** | **D_B_/L_S_** | **L_CP_/L_S_** | **L_CF_/L_CP_** |
| --- | --- | --- | --- | --- | --- | --- | --- |
| ***E. tetradactylum*** | ***ZJ*** | 0.254 | 0.168 | 0.685 | 0.247 | 0.157 | 1.68 |
|  | ***PA*** | 0.266 | 0.181 | 0.709 | 0.243 | 0.149 | 1.75 |
| ***E. rhadinum*** | ***ZJ*** | 0.243 | 0.197 | 0.647 | 0.255 | 0.169 | 1.742 |
|  | ***SA*** | 0.227 | 0.193 | 0.548 | 0.247 | 0.177 | 1.652 |
|  | ***JH*** | 0.234 | 0.152 | 0.561 | 0.241 | 0.166 | 1.703 |
|  | ***ZZ*** | 0.269 | 0.194 | 0.640 | 0.244 | 0.172 | 1.837 |

**L_H_/L_S_**: head length/standard length; **D_E_/L_H_**: eye diameter/head length; **D_CP_/L_CP_**: depth of caudal peduncle/length of caudal peduncle; **D_B_/L_S_**: body depth/standard length; **L_CP_/L_S_**: length of caudal peduncle/standard length; and **L_CF_/L_CP_**: length of caudal fin/length of caudal peduncle

**Table S7**. Principal component analysis (PCA) of morphometric variables *E. tetradactylum* and *E. rhadinum.*

| **morphometric variables** | ***E. tetradactylum*** | | | ***E. rhadinum*** | | |
| --- | --- | --- | --- | --- | --- | --- |
|  | **PC1** | **PC2** | **PC3** | **PC1** | **PC2** | **PC3** |
| **L_H_/L_S_** | 0.07305 | **-0.81379** | 0.16073 | 0.38248 | -0.14556 | **0.69152** |
| **D_E_/L_H_** | 0.24707 | 0.32531 | -0.65381 | -0.00309 | **0.67347** | 0.39044 |
| **D_CP_/L_CP_** | **0.55906** | 0.05402 | 0.14679 | **0.56505** | 0.21598 | -0.03541 |
| **D_B_/L_S_** | 0.04688 | 0.46772 | **0.72021** | 0.1037 | 0.66014 | -0.36945 |
| **L_CP_/L_S_** | **-0.57972** | 0.09558 | 0.01395 | -0.49438 | 0.19529 | 0.47763 |
| **L_CF_/L_CP_** | **0.53179** | -0.03319 | 0.07908 | 0.52844 | -0.06849 | 0.05898 |
| **Percentage of Variance** | 42.50% | 19.71% | 19.06% | 42.19% | 23.53% | 15.32% |
| **Cumulative** | 42.50% | 62.21% | 81.27% | 42.19% | 65.72% | 81.05% |

**L_H_/L_S_**: head length/standard length; **D_E_/L_H_**: eye diameter/head length; **D_CP_/L_CP_**: depth of caudal peduncle/length of caudal peduncle; **D_B_/L_S_**: body depth/standard length; **L_CP_/L_S_**: length of caudal peduncle/standard length; and **L_CF_/L_CP_**: length of caudal fin/ length of caudal peduncle

**Table S8-1**. Mahalanobis and Procrustes distances among populations of *E. tetradactylum* and *E. rhadinum.*

| **Population** | **Mahalanobis distances** | | | | | **Procrustes distances** | | | | |
| --- | --- | --- | --- | --- | --- | --- | --- | --- | --- | --- |
|  | **ERJH** | **ERSA** | **ERZJ** | **ERZZ** | **ETPA** | **ERJH** | **ERSA** | **ERZJ** | **ERZZ** | **ETPA** |
| **ERSA** | 2.6777 |  |  |  |  | 0.0276 |  |  |  |  |
| **ERZJ** | 5.5718 | 5.25 |  |  |  | 0.041 | 0.0336 |  |  |  |
| **ERZZ** | 5.7186 | 5.4544 | 4.8768 |  |  | 0.0477 | 0.0458 | 0.0413 |  |  |
| **ETPA** | 7.2411 | 6.2745 | 5.7297 | 6.3985 |  | 0.0696 | 0.055 | 0.0437 | 0.0631 |  |
| **ETZJ** | 4.3004 | 3.6127 | 3.2838 | 4.1637 | 5.0451 | 0.0356 | 0.0316 | 0.0244 | 0.0358 | 0.0509 |

**Table S8-2**. P-values from permutation tests (10000 permutation rounds) for Mahalanobis and Procrustes distances among populations of *E. tetradactylum* and *E. rhadinum.*

| **Population** | **P-values for Mahalanobis distances** | | | | | **P-values for Procrustes distances** | | | | | |
| --- | --- | --- | --- | --- | --- | --- | --- | --- | --- | --- | --- |
|  | **ERJH** | **ERSA** | **ERZJ** | **ERZZ** | **ETPA** | **ERJH** | **ERSA** | **ERZJ** | **ERZZ** | **ETPA** |  |
| **ERSA** | <.0001 |  |  |  |  | <.0001 |  |  |  |  |  |
| **ERZJ** | <.0001 | <.0001 |  |  |  | <.0001 | <.0001 |  |  |  |  |
| **ERZZ** | <.0001 | <.0001 | <.0001 |  |  | <.0001 | <.0001 | <.0001 |  |  |  |
| **ETPA** | <.0001 | <.0001 | <.0001 | <.0001 |  | <.0001 | <.0001 | <.0001 | <.0001 |  |  |
| **ETZJ** | <.0001 | <.0001 | <.0001 | <.0001 | <.0001 | <.0001 | <.0001 | <.0001 | <.0001 | <.0001 |  |
